# Distinct actions of testicular endocrine and lumicrine signaling on the proximal epididymal transcriptome

**DOI:** 10.1186/s12958-024-01213-x

**Published:** 2024-04-10

**Authors:** Daiji Kiyozumi

**Affiliations:** 1 Japan Science and Technology Agency, 7, Gobancho, Chiyoda-ku, Tokyo, 102-0076 Japan; 2 Research Institute of Environmental Medicine, Nagoya University, Furo-cho, Chikusa-ku, Nagoya, 464-8601 Japan; 3 Research Institute for Microbial Diseases, Osaka University, 3-2, Yamadaoka, Suita, Osaka 565-0871 Japan

**Keywords:** Lumicrine, Endocrine, Testis, Epididymis, Initial segment

## Abstract

The epididymal function and gene expression in mammals are under the control of the testis. Sex steroids are secreted from the testis and act on the epididymis in an endocrine manner. There is another, non-sex steroidal secreted signaling, named lumicrine signaling, in which testis-derived secreted proteins go through the male reproductive tract and act on the epididymis. The effects of such multiple regulations on the epididymis by the testis have been investigated for many genes. The recent development of high-throughput next-generation sequencing now enables us a further comparative survey of endocrine and lumicrine action-dependent gene expression. In the present study, testis-derived endocrine and lumicrine actions on epididymal gene expression were comparatively investigated by RNA-seq transcriptomic analyses. This investigation utilized experimental animal models in which testis-derived endocrine and/or lumicrine actions were interfered with, such as unilateral or bilateral orchidectomy. By bilateral orchidectomy, which interferes with both endocrine and lumicrine actions, 431 genes were downregulated. By unilateral orchidectomy, which also interferes with endocrine and lumicrine actions by the unilateral testis, but the endocrine action was compensated by the contralateral testis, 283 genes were downregulated. The content of such genes downregulated by unilateral orchidectomy was like those of lumicrine action-interfered efferent duct-ligation, *W*/*Wv*, and *Nell2*^−/−^ mice. When genes affected by unilateral and bilateral orchidectomy were compared, 154 genes were commonly downregulated, whereas 217 genes were specifically downregulated only by bilateral orchidectomy, indicating the distinction between endocrine and lumicrine actions on the proximal epididymal transcriptome. Comparative transcriptome analyses also showed that the expressions of genes emerging since Amniota were notably impacted by bilateral orchidectomy, unilateral orchidectomy, and lumicrine action-interfering treatments; the degree of influence from these treatments varied based on the evolutionary stage beyond Amniota. These findings unveil an evolutional transition of regulated gene expression in the proximal epididymis by two different testis-derived signaling mechanisms.

**Supplementary Information:**

The online version contains supplementary material available at 10.1186/s12958-024-01213-x.

## Introduction

The epididymis is a highly coiled epithelial duct constituting a part of the sperm transport route. After production in the testis, the testicular spermatozoa are transported through the efferent duct toward the epididymis, where sperm undergo further maturation of sperm functions such as motility and binding to oocytes necessary for their full fertilizing ability [1–4]. If the spermatozoa are not properly matured by the epididymis, they will not be able to acquire the cellular functions necessary for fertilization, eventually resulting in a significant decrease in male reproductive ability.

Such epididymal functions are mediated by the specific expressions of various genes. The epididymal expressions of specific genes are often responsible for the physiological functions of the epididymis and eventually the downstream sperm maturation [5, 6]. Interestingly, the epididymal gene expressions are known to be regulated by extra-epididymal or testicular factors. There are the endocrine and the non-endocrine signaling mechanisms, as signaling systems between the testis and the epididymis. In endocrine regulation, sex steroids originating from testicular Leydig cells reach the epididymis through the bloodstream. They act on epididymal cells by binding with androgen or estrogen receptors [7–12]. In non-endocrine regulation, secreted proteins synthesized by the testicular germ cells located inside the seminiferous tubule are secreted into the seminiferous fluid and reach the epididymis via the reproductive tract by the luminal flow [13–15]. The secreted proteins act on epididymal cells by binding to their receptors expressed on the cell surface of the epididymal luminal epithelium [15–17]. Since this type of secretion signaling between the testes and epididymis acts through the lumen, it has been referred to as “lumicrine signaling,” a terminology introduced by Barry T. Hinton [18]. Eventually, these testis-derived signals regulate the physiological functions of the epididymis by modifying gene expressions.

The testicular regulations of the epididymal cell functions and gene expressions can be investigated experimentally; in the early studies, when the testes were experimentally removed by orchidectomy (OD) or testis-epididymis luminal communication was interfered with by the efferent duct ligation (EDL), then the epididymal protein synthesis, which was monitored by the metabolic labeling using radioisotopes, was critically affected [19–21]. In addition to these OD or EDL-treated animals, gene-modified animals, in which the expression and function of endocrine or lumicrine signaling components are genetically ablated, are also available [8, 15–17, 22]. By using such animal models, various genes have been identified to be expressed in the epididymis in an endocrine action-dependent and/or lumicrine action-dependent manner [15, 16, 23–32].

Recently, it has become possible to examine gene expression by next-generation sequencing and analyze them not only by the expression levels of individual genes but with information about the structure, function, and evolution of gene products. Such data-driven analyses may allow for grouping genes based on their respective regulatory mechanisms or extracting the unknown features of regulated gene expression for gene groups classified according to specific criteria. In the present study, the gene expressions of the proximal epididymis, subjected to OD or lumicrine action-interfering treatments, were investigated through RNA sequencing (RNA-seq) and the obtained transcriptomes were subsequently evaluated to characterize the endocrine and lumicrine regulations of epididymal gene expression.

## Materials and methods

### Animals

B6D1F1 male mice were purchased from Japan SLC. Unilateral and bilateral ODs were performed as follows. Eight-week-old wild-type (WT) B6D1F1 males were unilaterally orchidectomized (*n* = 3), in which the contralateral untreated side served as control. Eight-week-old WT B6D1F1 males were bilaterally orchidectomized (*n* = 3) or sham-operated (*n* = 3), which served as controls. The initial segment (IS)-caput epididymides were isolated from the animals four weeks after OD or sham operation.

### Dissection of epididymis

The IS was dissected together with the caput and such a tissue dissection was indicated by the description “IS-caput” as described previously [16, 30]. This is because of the difficulty in dissecting IS separately from caput epididymides, especially in mice in which IS differentiation is ablated by the experimental treatments.

### RNA-seq

Total RNAs were isolated from the isolated IS-caput epididymides using RNeasy mini (Qiagen). On-column DNase treatment was performed during RNA purification using an RNase-free DNase set (Qiagen). The amount of RNAs was determined by absorbance at 260 nm. The RNA-seq of epididymal transcripts was performed as follows: libraries for sequencing were prepared from isolated RNAs using a TruSeq stranded mRNA sample prep kit (Illumina, #20,020,594) and sequenced on a NovaSeq6000 (Illumina) using 101 bp single-ended mode. The mapping of the obtained sequence reads onto a mouse reference genome (mm10) was performed using TopHat ver. 2.1.1 [33]. To calculate fragments per kilobase of exon per million mapped reads (FPKM) values for each gene, Cufflinks ver. 2.2.1 was used [34]. The obtained RNA-seq data have been deposited in the Gene Expression Omnibus database under the accession code GSE247764.

### Transcriptome analyses

The IS-caput epididymal transcriptomes of unilateral OD, bilateral OD, and their controls were comparatively analysed. The IS-caput epididymal transcriptomes of EDL (EDL performed at 10 weeks old and the ipsilateral epididymis was at 14 weeks old) [30], *W*/*Wv*, a *Kit* compound heterozygous mutant (14 weeks old) [30], and *Nell2*^−/−^ (14 weeks old) [15] mice were also used for comparison (datasets are publicly available from the NCBI Gene Expression Omnibus website (https://www.ncbi.nlm.nih.gov/geo/). The transcriptome data were incorporated into Microsoft Excel software for further analysis. Gene ontology (GO) information was obtained from the Mouse Genome Informatics website (https://www.informatics.jax.org/vocab/gene_ontology).

Mouse genes were classified into nine classes, i.e., Craniata, Gnathostomata, Teleostomi, Tetrapoda, Amniota, Mammalia, Theria, Euthelia, and others according to the emergence of genes during vertebrate evolution, using the information provided by NCBI Gene (https://www.ncbi.nlm.nih.gov/gene).

### Drawings

Schematic drawings were generated using Microsoft PowerPoint (Microsoft Corporation). Plot representations, heatmap representations, and bar graphs were generated using Microsoft Excel 2019 (Microsoft Corporation).

### Statistical analysis

Two-tailed *t*-tests under the assumption of unequal variances were performed using Microsoft Excel 2019.

## Results

### Comparative transcriptome analyses of orchidectomized mouse proximal epididymis

**Fig. 1:**
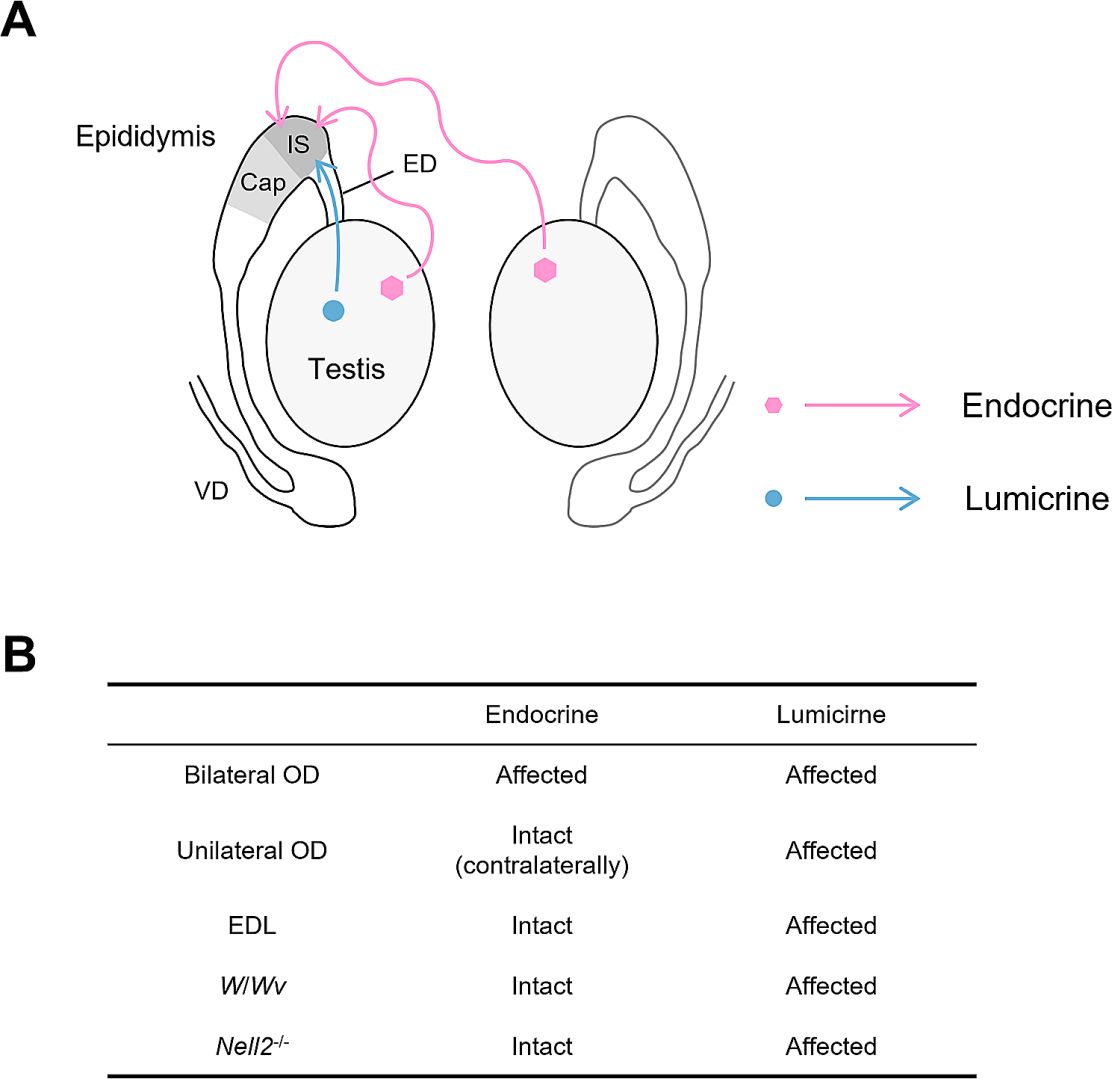
Testis-derived endocrine and lumicrine actions on the epididymis. **(A)** A schematic representation of testis-derived endocrine and lumicrine actions on the epididymis. IS, initial segment; Cap, caput; ED, efferent duct; VD, vas deferens. Blue arrows indicate the lumicrine action through the male reproductive tract. Red arrows indicate the endocrine action through the bloodstream. **(B)** A summary of experimental conditions and their effects on endocrine and lumicrine actions

A schematic representation of endocrine and lumicrine actions from the testis to the epididymis is represented in Fig. 1. In the bilateral OD, both testes are removed and both endocrine and lumicrine actions are ablated. In the unilateral OD, the unilateral testis is removed, then the ipsilateral lumicrine and endocrine actions are ablated but the contralateral testis-derived endocrine action is expected to be still active. In the EDL, only lumicrine is ablated because the testis-epididymis luminal connection is interfered with. Since *W*/*Wv* and *Nell2*^−/−^ mice lack germ cells that secrete lumicrine ligands and lumicrine ligand NELL2, respectively, only lumicrine but not endocrine signaling is dysfunctional in these animals [15].

**Fig. 2:**
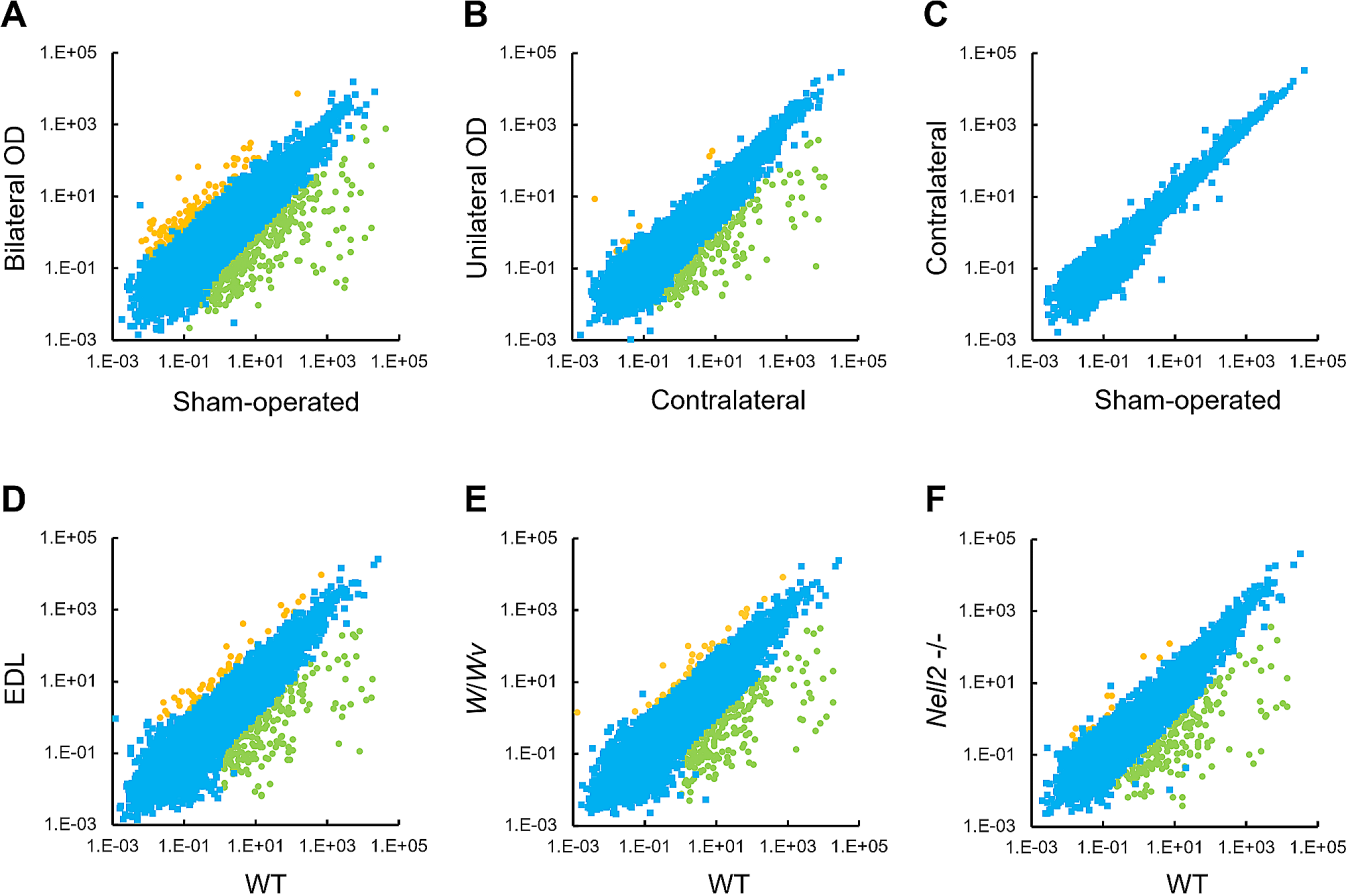
RNA-seq analyses of orchidectomized and lumicrine signaling-deficient IS-caput epididymis. **A**–**C**, RNA-seq of sham-operated control vs. bilateral OD (**A**), contralateral side control vs. ipsilateral side in unilateral OD (**B**), sham-operated control vs. contralateral side control in unilateral OD (**C**). D-F, RNA-seq of WT vs. EDL (**D**), WT vs. *W*/*Wv* (**E**), and WT vs. *Nell2*^−/−^ (**F**). FPKM values are plotted. Statistically significantly downregulated (fold change < 0.1, and t-test *P* < 0.05) and upregulated (fold change > 10, and t-test *P* < 0.05) genes are represented in green and yellow, respectively

The unilateral or bilateral OD was performed at 8 weeks old for 4 weeks and the IS-caput epididymides were isolated for RNA isolation and the subsequent transcriptome analysis. For unilateral OD, contralateral epididymides were used as controls. For bilateral OD, the epididymides of sham-operated animals were used as controls. The obtained RNA-seq results were summarized in Supplementary Data File 1. The IS-caput epididymal gene expression in the orchidectomized animals and those in their control animals were plot-represented (Fig. 2A-B). The unilateral OD did not significantly affect the gene expression of the contralateral side epididymis, as evidenced by the comparison between the sham-operated control and the contralateral side control of unilateral OD (Fig. 2C). For the comparative study, the IS-caput transcriptomes of EDL, *W*/*Wv*, and *Nell2*^−/−^ animals, all of which were previously done by the author based on the experimental procedure identical to that employed in the present study [15, 30], were also shown (Fig. 2D–F).

In the bilateral OD treatment, 431 genes were significantly downregulated (Table 1). Among such genes downregulated in bilateral OD, 27.8 ∼ 41.5% are common with those with unilateral OD (154 genes), EDL (179 genes), *W*/*Wv*, (165 genes), and *Nell2*^−/−^ (120 genes). The proportion of unique genes whose expression was significantly downregulated in the bilateral OD animals was 50.3% (217 genes), which was very high compared with those in unilateral OD (14.8%), EDL (22.1%), *W*/*Wv* (9.0%), and *Nell2*^−/−^ (10.4%) (Table 1). In the unilateral OD, 283 genes were significantly downregulated (Table 1). Among them, 49.1 ∼ 77.0% are common with those with bilateral OD (154 genes), EDL (218 genes), *W*/*Wv*, (187 genes), and *Nell2*^−/−^ (139 genes). The proportion of unique genes whose expression was significantly downregulated in the unilateral OD animals was 14.8% (42 genes). Thus, while many genes were commonly downregulated among experimentally treated animals including bilateral OD and unilateral OD, there were a considerable number of genes specifically downregulated by bilateral OD.

**Table 1:** Comparison of downregulated genes (fold change < 0.1, and t-test *P* < 0.05) in orchidectomized and lumicrine signaling-deficient mouse IS-caput epididymides

	Fold change < 0.1 *P* < 0.05	Unique	Common with				
			Bilateral OD	Unilateral OD	EDL	W/Wv	Nell2^−/−^
Bilateral OD	431	217 (50.3%)		154 (35.7%)	179 (41.5%)	165 (38.3%)	120 (27.8%)
Unilateral OD	283	42 (14.8%)	154 (54.4%)		218 (77.0%)	187 (66.1%)	139 (49.1%)
EDL	476	105 (22.1%)	179 (37.6%)	218 (45.8%)		311 (65.3%)	188 (39.5%)
*W*/*Wv*	366	33 (9.0%)	165 (45.1%)	187 (52.2%)	311 (85.0%)		187 (51.1%)
*Nell2* ^−/−^	231	24 (10.4%)	120 (52.9%)	139 (60.2%)	188 (81.4%)	187 (81.0%)	

**Fig. 3:**
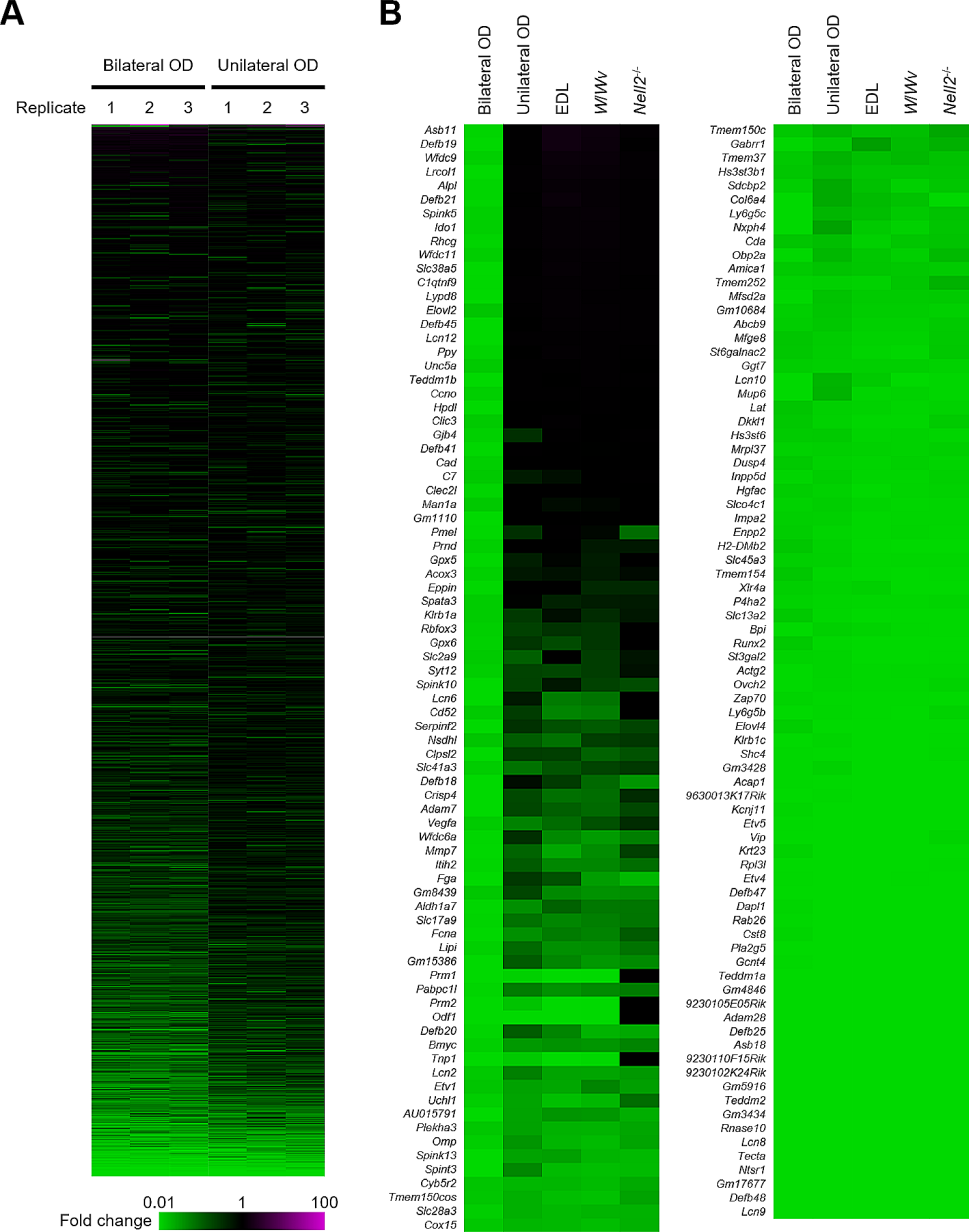
Comparative representation of genes downregulated in lumicrine signaling-deficient and endocrine signaling-deficient mouse IS-caput epididymides (**A**) Fold change of gene expressions in bilateral OD (*n* = 3) and unilateral OD (*n* = 3). Green and magenta represent downregulation and upregulation, respectively. (**B**) Genes downregulated in bilateral OD IS-caput epididymis (fold change < 0.1, and t-test *P* < 0.05) compared with fold changes in unilateral OD, EDL, *W*/*Wv*, and *Nell2*^−/−^ IS-caput epididymis. Average values are shown. Color indications are the same as in panel A

The fold changes of gene expression in the bilateral and unilateral OD-treated IS-caput epididymides were shown by heatmap representation (Fig. 3A). For genes significantly downregulated in bilateral OD-treated IS-caput epididymis (fold change < 0.1, and *t*-test *P* value < 0.05), their expression fold changes were compared with those of unilateral OD, EDL, *W*/*Wv*, and *Nell2*^−/−^ animals (Fig. 3B and Supplementary Data File 2). Among genes significantly downregulated in the bilateral OD animals, there are many genes not affected or only moderately downregulated in the unilateral OD, EDL, *W*/*Wv*, and *Nell2*^−/−^ animals, endorsing the above observation that a larger number of genes were downregulated in the bilateral OD mice compared with unilateral OD, EDL, *W*/*Wv*, and *Nell2*^−/−^ ones.

For upregulated genes, there are 196 and 69 genes upregulated by bilateral OD and unilateral OD, respectively (Table 2). However, among such upregulated genes, only 1 ∼ 3 genes were common between genes upregulated in the IS-caput epididymis of EDL, *W*/*Wv*, or *Nell2*^−/−^ mice, implying that the observed upregulations are resulting from experimental variation than from a common mechanism.

**Table 2:** Comparison of upregulated genes (fold change > 10, and t-test *P* < 0.05) in orchidectomized and lumicrine-deficient mouse IS-caput epididymides

	Fold change > 10 *P* < 0.05	Unique	Common with				
			Bilateral OD	Unilateral OD	EDL	W/Wv	Nell2^−/−^
Bilateral OD	196	180 (91.8%)		7 (3.6%)	4 (2.0%)	3 (1.5%)	5 (2.6%)
Unilateral OD	69	57 (82.6%)	7 (10.1%)		2 (2.9%)	1 (1.4%)	2 (2.9%)
EDL	107	66 (61.7%)	4 (3.7%)	2 (1.9%)		33 (30.8%)	6 (5.6%)
*W*/*Wv*	67	31 (46.3%)	3 (4.5%)	1 (1.5%)	33 (49.3%)		2 (3.0%)
*Nell2* ^−/−^	67	54 (80.6%)	5 (7.5%)	2 (3.0%)	6 (9.0%)	2 (3.0%)	

Collectively, these results indicate that unilateral OD, EDL, *W*/*Wv*, and *Nell2*^−/−^ animals are similar whereas the bilateral OD animals are rather unique in their gene downregulation. Hereafter in the present study, unilateral OD is therefore treated as a variation of the lumicrine action-interfering treatments (see also Discussion).

### The feature of gene products downregulated in endocrine and/or lumicrine actions-interfered mouse epididymis

The expression of many genes was influenced differently by the bilateral OD and lumicrine action-interfering treatments. To investigate whether there are common characteristics shared among such genes whose expressions were affected, genes were classified based on their function or their evolution. Subsequently, the total expressions of genes classified in such a manner were compared between the experimental groups.

**Fig. 4:**
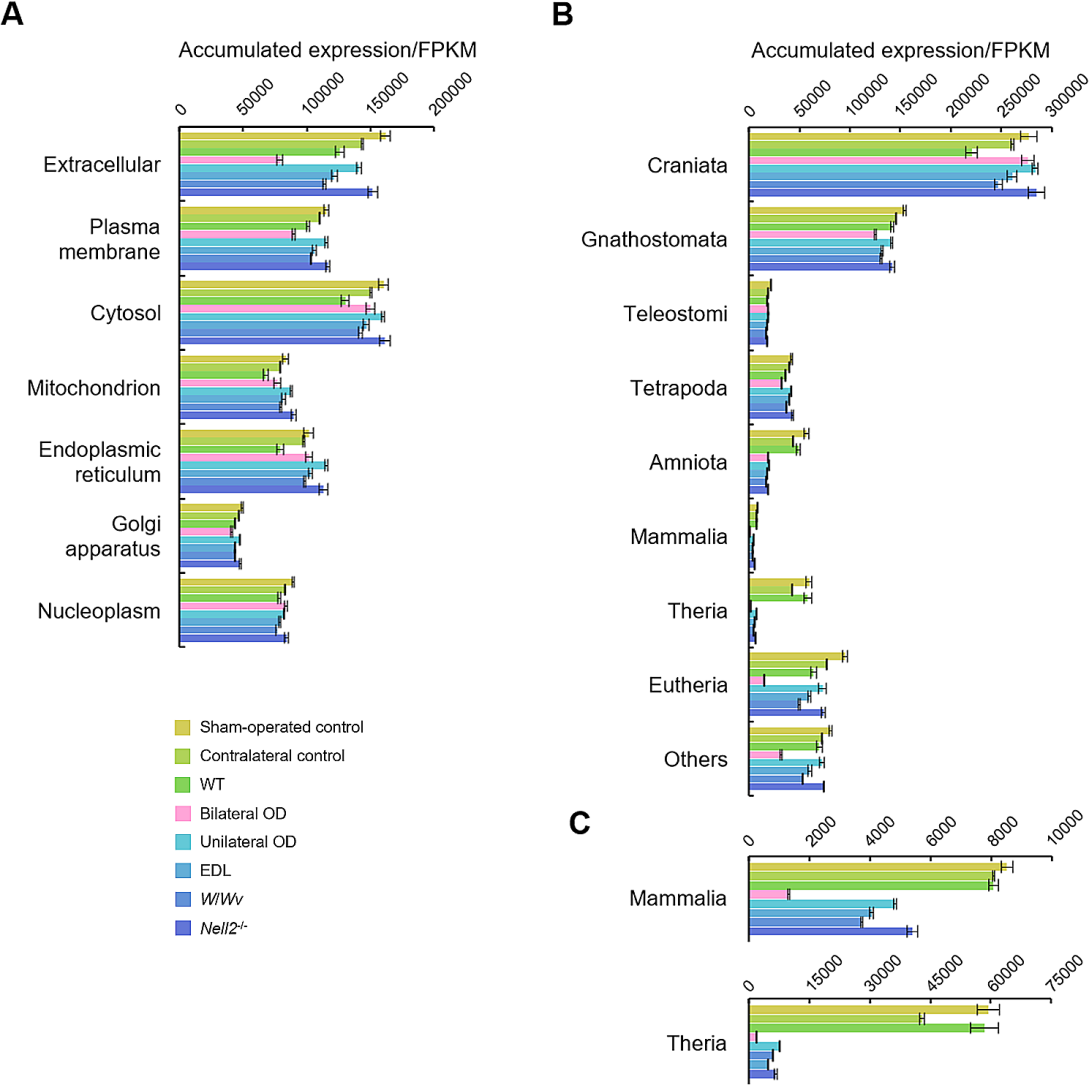
Expression of genes classified based on the properties of resulting proteins. Gene expressions in sham-operated control, contralateral control, WT, bilateral OD, unilateral OD, EDL, *W*/*Wv*, and *Nell2*^−/−^ IS-caput epididymis. (**A**) Genes are classified by subcellular localization using GO information. The expression levels of genes were accumulated for each group. (**B**) Genes are classified according to their evolution. The expression levels of genes were accumulated for each group. (**C**) Magnified representations of Mammals and Theria in panel B. All values are shown as mean ± S.E.M (*n* = 3)

Using GO information, genes were selected based on the localization of the encoded proteins (extracellular, plasma membrane, cytosol, mitochondrion, endoplasmic reticulum, Golgi apparatus, and nucleoplasm). Gene expression was then accumulated for each classification group and compared between experimental groups (Fig. 4A and Supplementary Data File 3). An apparent reduction by bilateral OD was observed in GO “extracellular” genes. In other GO classifications, prominent downregulation or upregulation of the accumulated gene expression was not recognized in the specific experimental group.

Transcriptomes can be analyzed not only based on gene functions but also based on gene evolution. Genes were therefore classified into nine evolutional classes, i.e., Craniata, Gnathostomata, Teleostomi, Tetrapoda, Amniota, Mammalia, Theria, Euthelia, and others (evolutionally newer genes), and the accumulated gene expressions for each class were compared between experimental groups (Fig. 4B and C and Supplementary Data File 4). There was no critical difference in the accumulated expression of genes acquired before Tetrapoda between the experimental groups, whereas apparent reductions in the accumulated expression by the experimental treatments were observed in genes acquired since Amniota. The accumulated expressions of Amniota-specific genes were reduced in mice with bilateral OD and mice with lumicrine action-interfering treatments such as unilateral OD, EDL, *W*/*Wv*, and *Nell2*^−/−^ mice. For Mammalia- and Theria-specific genes, their accumulated expressions were also reduced similarly in the unilateral OD, EDL, *W*/*Wv*, and *Nell2*^−/−^ animals, whereas the reduction in the bilateral OD was even greater. Decreased expression was also observed in the genes of For Euthelia-specific and evolutionally newer genes, the reduction of accumulated gene expression was apparent only in the bilateral OD animals but not prominent in those of unilateral OD, EDL, *W*/*Wv*, and *Nell2*^−/−^ animals. Thus, the expressions of genes acquired since Amniota were affected by bilateral OD and other lumicrine action-interfering treatments but to a different extent.

**Fig. 5:**
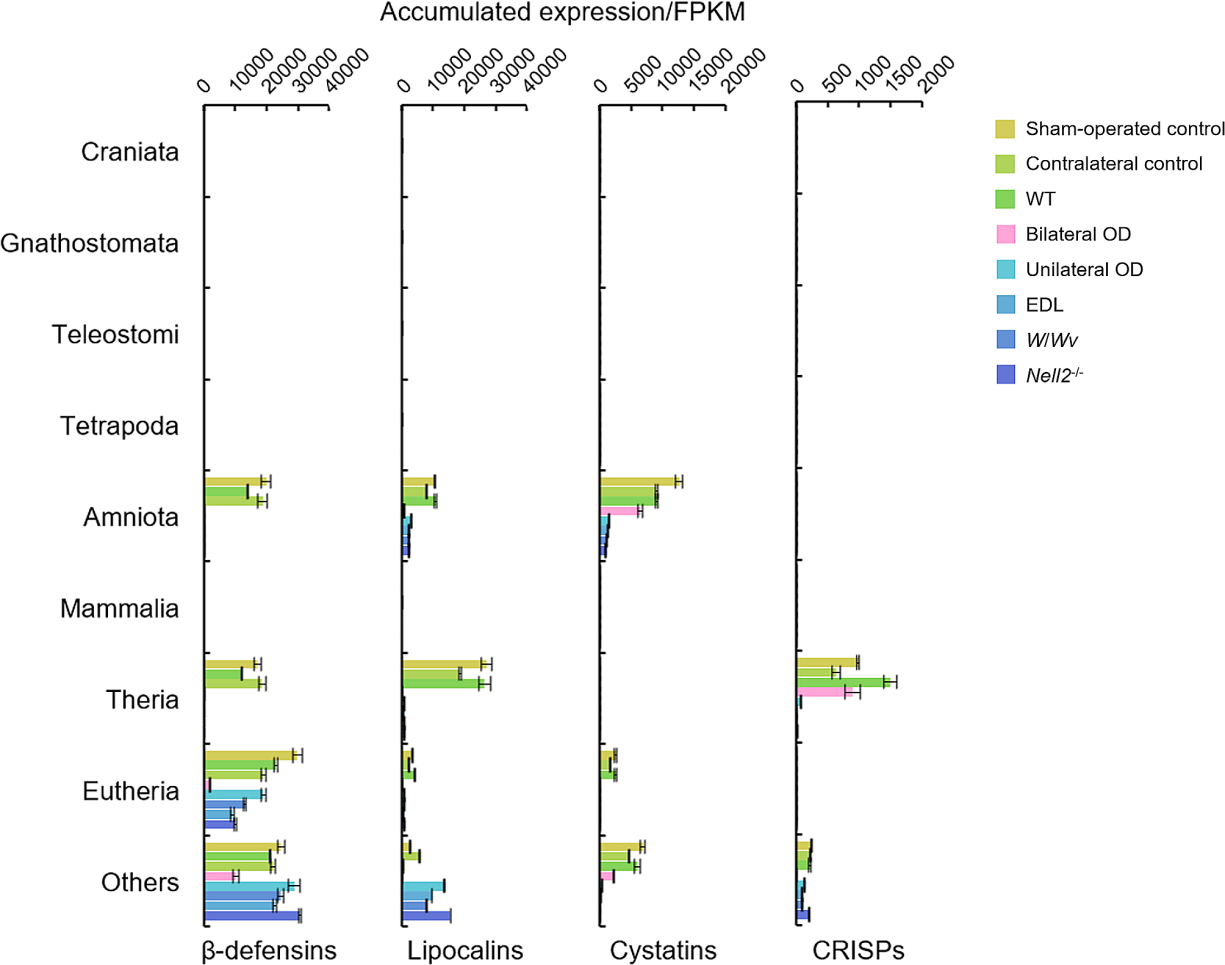
Expression of gene families abundantly expressed in the epididymis. The accumulated expression levels of genes encoding β-defensins, lipocalins, cystatins, and CRISPs in sham-operated control, contralateral control, WT, bilateral OD, unilateral OD, EDL, *W*/*Wv*, and *Nell2*^−/−^ IS-caput epididymis are shown according to their evolution. All values are shown as mean ± S.E.M (*n* = 3)

There are several protein families known to be specifically expressed in the epididymis such as β-defensins [35, 36], cystatins [37, 38], cysteine-rich secretory proteins (CRISPs) [39–41], and lipocalins [42, 43]. The regulated expressions of genes encoding such protein families were also investigated from the evolutional aspect (Fig. 5). All genes encoding these protein families have emerged since Amniota. The expressions of Amniota-specific β-defensin, cystatin, and lipocalin genes are downregulated by either or both bilateral OD and lumicrine action-interfering treatments, whereas Amniota-specific CRISP genes were not expressed in both control and experimentally treated IS-caput epididymis. Also, the expressions of genes β-defensin, lipocalin, cystatin, and CRISP genes emerged since Theria were affected by both bilateral OD and lumicrine action-interfering treatments. Collectively, bilateral OD and lumicrine action-interfering treatments differently but critically affected the IS-caput epididymal expressions of genes acquired since Amniota.

## Discussion

There are non-autonomous mechanisms that regulate epididymal cell differentiation and gene expression. By observing changes in the epididymis following OD and EDL, endocrine and lumicrine actions have been identified as secretory signaling mechanisms originating from this testis [13, 14, 44]. The actions of these two regulatory mechanisms on the epididymal gene expression have been investigated for many genes but have not been investigated comprehensively at the genomic level. However, recent advances in the analytical capabilities of next-generation sequencing have made it possible to carry out comparative genome-wide expression analyses with higher precision and comprehensiveness than has been possible in the past. In the present study, the features of endocrine and lumicrine actions to regulate epididymal gene expression were examined by transcriptome analyzes. In the bilateral OD, the testicular endocrine and lumicrine actions are completely ablated. In the unilateral OD, the endocrine and lumicrine actions originating from the ipsilateral testis on the epididymis are ablated, but the endocrine action is still compensated by that from the contralateral testis. The comparative transcriptome analyses confirmed that the gene expression profile of unilateral OD is different from that of bilateral OD but rather like those of the lumicrine action-interfered animals such as EDL, *W*/*Wv*, and *Nell2*^−/−^ mice (Figs. 2 and 3; Table 1). Thus, as briefly described in the Result section, unilateral OD is concluded to be a variation of the lumicrine action-interfering treatments.

### Overview of testicular regulations of epididymal gene expression

The preceding studies unveiled that among genes expressed in the epididymis, some are regulated by the testicular endocrine and/or lumicrine signaling [8, 15, 16, 23, 25, 27–31, 45–214]. In the present study, genes whose expressions were regulated by testicular endocrine and/or lumicrine actions were explored further by comparing RNA-seq data obtained from bilateral or unilateral OD, EDL, *W*/*Wv*, and *Nell2*^−/−^ mouse epididymis. Genes whose expression is reduced by experimental treatment are especially important because their gene expression is positively regulated under physiological conditions. Such downregulated genes can be classified into several groups: i, genes downregulated by lumicrine action-interfering treatments, and therefore affected equally also by bilateral OD; ii, genes not affected by lumicrine action-interfering treatments, but downregulated only by bilateral OD; iii, genes moderately downregulated by lumicrine action-interfering treatments and further by bilateral OD. Not all genes downregulated by unilateral OD are solely regulated by lumicrine signaling. The differentiation and associated gene expression of the IS epididymis require androgen action even if the luminal communication between testis and epididymis is intact; in *Rnase10-cre*; *Ar*^loxP^ mice, in which androgen receptors are conditionally knocked out in the proximal epididymis, the IS differentiation and associated gene expression are inhibited [8]. Therefore, the expression of genes downregulated by lumicrine action-interfering treatment is also regulated in a concerted way by lumicrine and endocrine signaling mechanisms. Collectively, the epididymal expression of individual genes is regulated not solely by either endocrine or lumicrine mechanisms, but rather to varying degrees.

### Evolution of endocrine and lumicrine regulation of the epididymis by the testis

**Fig. 6:**
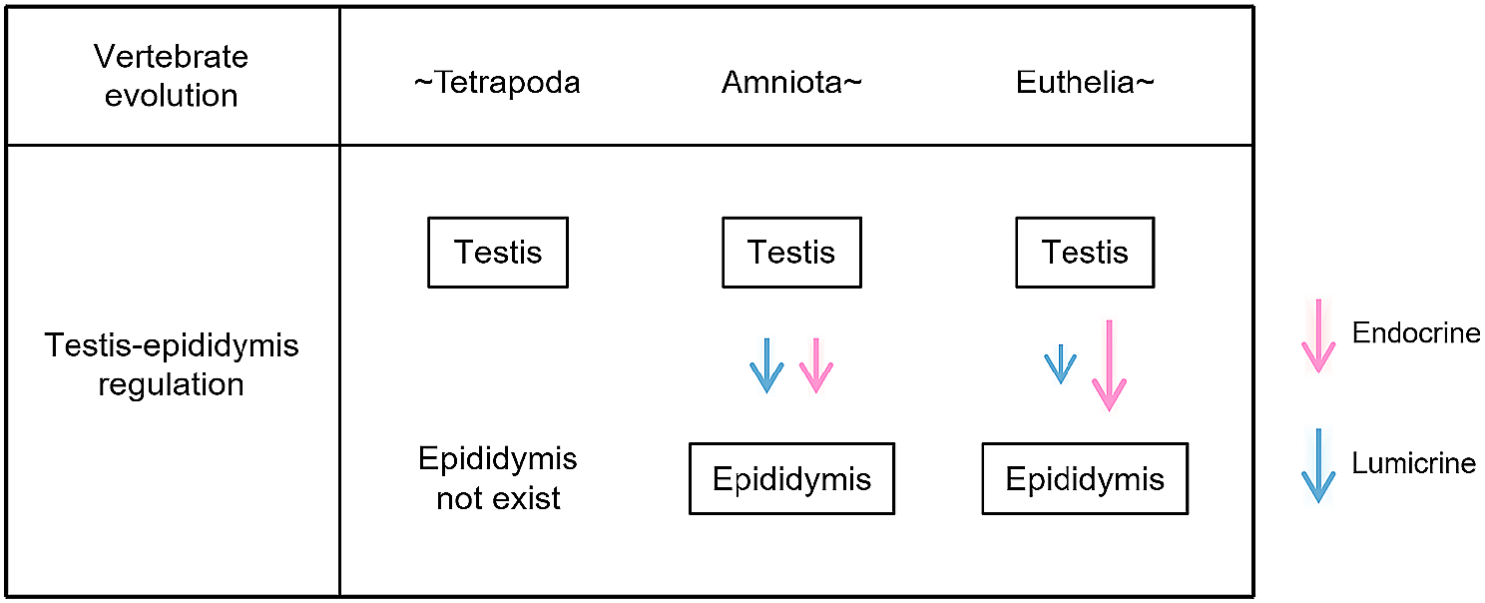
A hypothesis for the testis-epididymis secreted signaling during evolution. Before Amniota, the epididymis and therefore testis-epididymis secreted signaling did not exist. In Amniota, in which the epididymis had developed from the mesonephros, testis-derived endocrine and lumicrine regulation of epididymal gene expression (represented by pink and blue arrows, respectively) emerged. Such contributions by endocrine or lumicrine action to the epididymal gene expression (represented by the size of arrows) can alter along evolution

A variety of information for biological processes can be extracted by analyzing transcriptomes. In the present study, the transcriptomes were analyzed further based on the subcellular localization of gene products and gene evolution. Since such gene characterizations were based on the resulting proteins, genes encoding small non-coding RNAs, which are also enriched in the epididymis [215], were not analyzed in the present study. A comparison of the expression of genes selected based on GO protein localization information showed downregulation of GO “extracellular” genes by bilateral OD, indicating that the induction of extracellular proteins is one of the major targets of testicular endocrine signaling. On the other hand, expression comparisons of genes classified based on vertebrate evolutionary information showed there are selective regulations of gene expression by lumicrine and endocrine signaling mechanisms according to the evolutionary stage. The lumicrine signaling regulated the expression of genes acquired in Amniota, which corresponds to the early post-land expansion in the vertebrate evolutionary classification criteria adopted in this study. Since Amniota, regulation by both lumicrine and endocrine actions was found for genes acquired in Mammalia and Theria. For genes acquired evolutionarily more recently after Euthelia, only endocrine regulation was apparent. The expressions of several epididymis-specific genes such as encoding β-defensins, cystatins, CRISPs, and lipocalins were also specifically regulated by the testicular endocrine and lumicrine actions since Amniota. These findings suggest a possibility that testicular endocrine and lumicrine regulation of epididymal gene expression was active since the establishment of the epididymis in the Amniota but the extent of their contribution has been varying in the later evolution (Fig. 6). Among the genes acquired before Tetrapoda, there are genes such as *Adam28*, *Etv1*, *Etv4*, *Etv5*, *Mfge8*, and *Ovch2* whose epididymal expressions are regulated by endocrine and/or lumicrine signaling [15, 24, 216]. However, in contrast to the genes acquired after Amniota, the total expressions of genes acquired before Tetrapoda were not critically affected by the testicular endocrine and lumicrine regulations. These observations suggest a possibility that genes acquired before Tetrapoda function rather as housekeeping genes in the proximal epididymis whereas genes acquired after Amniota function as those responsible for epididymis-specific functions. The evolution of the epididymis has been investigated mainly by comparative anatomy [217]. Although the findings in the present study do not clarify the genetic mechanisms of epididymis formation, they will provide new insights into how epididymal gene expressions have been regulated by testicular endocrine and lumicrine actions through vertebrate evolution.

In conclusion, the present study has unveiled the different characteristics of the endocrine and lumicrine actions on the regulation of epididymal gene expression.

### Electronic supplementary material

Below is the link to the electronic supplementary material.

Supplementary Material 1

Supplementary Material 2

Supplementary Material 3

Supplementary Material 4

## Data Availability

All transcriptome data supporting the results of this study are available at the NCBI GEO under accession numbers GSE247764 (unilateral OD, bilateral OD, and Nell2-/-) and GSE232898 (W/Wv and EDL).

## Declarations

### Submission declaration and verification

The authors declare that this manuscript has not been published previously and is not under consideration for publication elsewhere.
